# Amber from the Triassic to Paleogene of Australia and New Zealand as exceptional preservation of poorly known terrestrial ecosystems

**DOI:** 10.1038/s41598-020-62252-z

**Published:** 2020-04-02

**Authors:** Jeffrey D. Stilwell, Andrew Langendam, Chris Mays, Lachlan J. M. Sutherland, Antonio Arillo, Daniel J. Bickel, William T. De Silva, Adele H. Pentland, Guido Roghi, Gregory D. Price, David J. Cantrill, Annie Quinney, Enrique Peñalver

**Affiliations:** 1 School of Earth, Atmosphere and Environment, 9 Rainforest Walk, Monash University, Clayton, Victoria 3800 Australia; 2 Department of Palaeobiology, Swedish Museum of Natural History, Box 50007, S-104 05 Stockholm, Sweden; 3 Departamento de Zoología y Antropología Física, Facultad de Biología, Universidad Complutense, 28040 Madrid, Spain; 4 Australian Museum, 1 William Street, Sydney, New South Wales 2000 Australia; 5 Faculty of Science, Engineering and Technology, Swinburne University of Technology, John St, Hawthorn, Victoria 3122 Australia; 6 Institute of Geosciences and Earth Resources, CNR, Padova, Italy; 7 School of Geography, Earth and Environmental Sciences (Faculty of Science and Engineering), Plymouth University, Room 105, Fitzroy, Drake Circus, Plymouth, Devon PL4 8AA UK; 8 Royal Botanic Gardens Victoria, South Yarra, Victoria 3141 Australia; 9 Department of Geoscience at the University of Calgary in Calgary, Alberta, T2N 1N4 Canada; 10 Instituto Geológico y Minero de España (Museo Geominero), 46004 Valencia, Spain

**Keywords:** Palaeontology, Biogeography, Taxonomy

## Abstract

The Northern Hemisphere dominates our knowledge of Mesozoic and Cenozoic fossilized tree resin (amber) with few findings from the high southern paleolatitudes of Southern Pangea and Southern Gondwana. Here we report new Pangean and Gondwana amber occurrences dating from ~230 to 40 Ma from Australia (Late Triassic and Paleogene of Tasmania; Late Cretaceous Gippsland Basin in Victoria; Paleocene and late middle Eocene of Victoria) and New Zealand (Late Cretaceous Chatham Islands). The Paleogene, richly fossiliferous deposits contain significant and diverse inclusions of arthropods, plants and fungi. These austral discoveries open six new windows to different but crucial intervals of the Mesozoic and early Cenozoic, providing the earliest occurrence(s) of some taxa in the modern fauna and flora giving new insights into the ecology and evolution of polar and subpolar terrestrial ecosystems.

## Introduction

**Figure 1:**
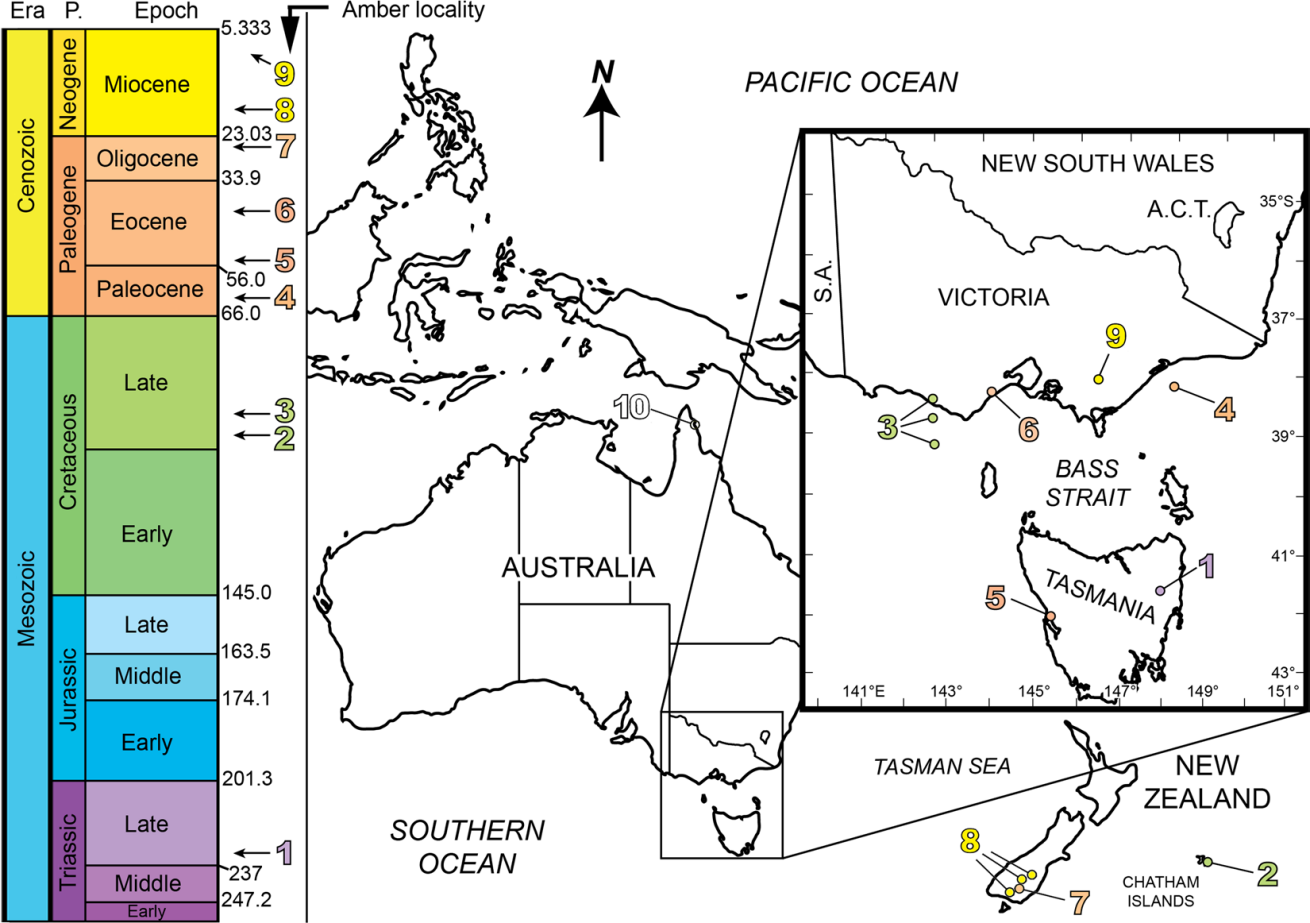
Map of Late Triassic to early Paleogene fossiliferous amber localities of Australia and Zealandia, Southern Pangea and Southern Gondwana. (**1**) Late Triassic: Fingal Coal Measures (Carnian Stage), Tasmania. (**2**) Late Cretaceous: Tupuangi Formation (upper Cenomanian of Chatham Islands), eastern Zealandia. (**3**) Late Cretaceous: Flaxman and Waarre formations of Victoria (Turonian Stage). (**4**) Early Paleocene: Barracouta-1 well, Latrobe Group, Gippsland Basin. (**5**) Early Eocene: Macquarie Harbour Formation, Strahan, Tasmania. (**6**) Late middle Eocene: A Group Coal Seam, Anglesea Coal Measures, Victoria. (**7**) Late Oligocene: Pomahaka, Southland, New Zealand^9^. (**8**) Early Miocene: three localities in Southland, New Zealand^9^. (**9**) Mio-Pliocene: Latrobe Valley Coal Measures, Victoria. (**10**) Uncertain aged (Paleogene or younger) pieces of amber from Cape York Peninsula (apparently float pieces). A.C.T. = Australian Capital Territory; P. = Period; S.A. = South Australia. Geochronological ages from^47^. Map generated using Adobe Illustrator CC2018 (www.adobe.com) and derived from sketching the region from Google Earth (www.earth.google.com).

Amber, or ancient tree resin, is valued most highly in science as an exceptional preservation medium for small organisms as fossil bioinclusions. In paleontology, diverse animals, plants and microorganisms have the potential of being preserved in three dimensions in the finest of detail. Worldwide, ambers have been recorded dominantly in upper Mesozoic to lower Cenozoic rocks from Northern Hemisphere and Northern Gondwana localities, but only one southern high latitude occurrence of microorganisms and microbe-like inclusions in amber has been published from the early Late Cretaceous (Turonian) in the Flaxman and Waarre formations of southern Victoria, Australia^1^. Other Late Cretaceous ambers have recently been reported from the Chatham Islands, New Zealand^2^, representing internal plant resin canals (no exuded amber), and small to minute amber fragments have been reported from the early Paleogene (early Eocene) of western Tasmania^3^, mid-Paleogene of Victoria^4^ and strandline deposits of the southern coast of Australia from Victoria to the west coast^5^ (Fig. 1). However, no preserved animals or plants have yet been found. Neogene ambers have been reported from the Mio–Pliocene Australian Latrobe Valley Coal, cropping out near Yallourn, Allendale and also Lal Lal in Victoria in Australia^6,7^ (Fig. 1). Earlier reports of Cretaceous amber sourced from the Wonthaggi Coal Mine^7^ have proved to be anomalous and not from the Mesozoic or early Cenozoic^1^. Amber from Cape York, far northern Queensland, Australia, is under study to establish if it is autochthonous or allochthonous (see discussion in Supplementary Text). New Zealand ambers have been reported from the latest Paleogene and early Neogene, such as the early Miocene of the Gore Lignite Measures, Southland^7–9^. In terms of northern Gondwana amber localities, these are sparse and include Brazil, Argentina, Peru, South Africa, and the Congo, but are low- to mid-paleolatitude Gondwanan sites only^10–13^. New, high southern paleolatitude localities and associated significant bioinclusions are the focus of this paper. These biostratigraphically well constrained, *in situ* amber sites with associated fossils are described below in sequence from oldest (Late Triassic) to youngest (mid-Paleogene), followed by the new records of animal, plant and fungus inclusions for Southern Pangea and Southern Gondwana.

## Results

### Triassic amber

**Figure 2:**
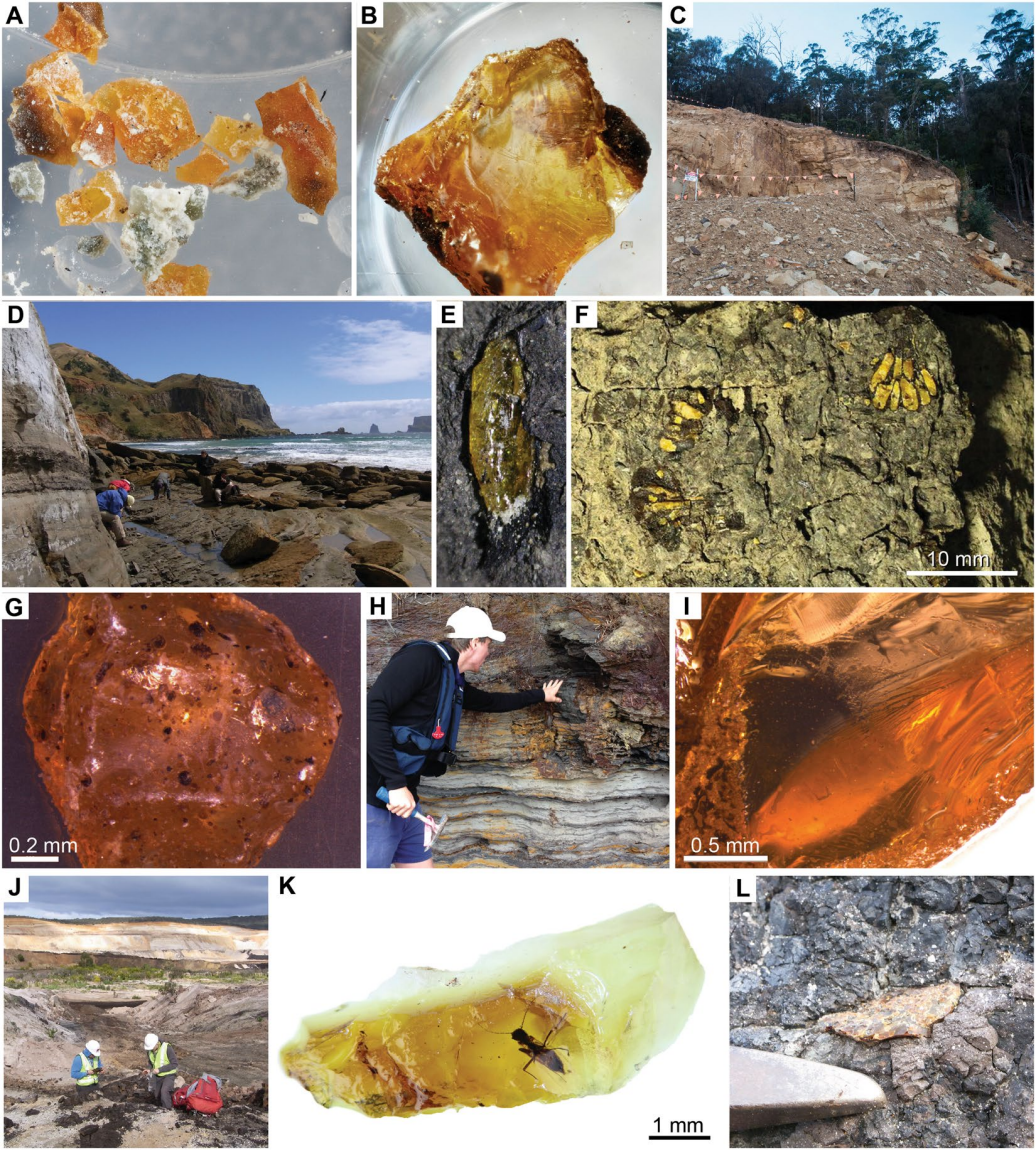
Late Triassic to early Paleogene ambers of Southern Pangea and Southern Gondwana with representative amber specimens and site images from the Late Triassic, Late Cretaceous and early Paleogene. (**A** to **C**) Fingal Valley, northeastern Tasmania, Fingal Valley Coal Measures, Carnian, Upper Triassic. (**A**) Several amber pieces <2 mm from the coal measures. (**B**) 2 mm piece of amber with preserved bark and microbe-like inclusions. (**C**) Photograph of Fingal Valley Triassic amber site. (**D** to **F**) Waihere Bay, western Pitt Island, Chatham Islands, Tupuangi Formation, upper Cenomanian, lower Upper Cretaceous. (**D**) Conducting fieldwork in the richly fossiliferous sections of the southern Waihere Bay. (**E**) Well preserved *in situ* amber droplet 3.2 mm long. (**F**) Seed cone scales of *Protodammara reimatamoriori* Mays & Cantrill, 2019, image depicting *in situ* resin canals within the dispersed ovuliferous complexes. (**G** to **I**) Strahan, Macquarie Harbour, Macquarie Harbour Formation, Western Tasmania, lower Eocene. (**G**) Inclusion**-**rich amber piece. (**H**) Section of Macquarie Harbour Formation along Macquarie Harbour near Strahan with fossiliferous strata. William De Silva for scale. (**I**) Clear, glassy amber specimen. (**J** to **L**) Anglesea Coal Measures, Alcoa Mine, Victoria, Australia, upper middle Eocene. (**J**) Collecting samples at main amber site. (**K**) Piece of clear yellow Anglesea amber with inclusion of new dipteran (Ceratopogonidae). (**L**) *In situ* amber piece at primary site with Estwing rock pick for scale.

Previously unrecorded, the oldest Southern Pangea amber occurs in the Upper Triassic terrestrial deposits of the Fingal Valley Coal Measures (‘Fingal Tier’) of the Upper Parmeener Supergroup of Tasmania, where it was discovered in 2015 (Figs. 2A,C and S1) (see also the Supplementary Text). The amber is found as small (≤1.5 mm long), mostly clear fragments with inclusions representing bark fragments, plant pieces, miniscule organic debris, and microbe-like inclusions within the top of the sequence in Unit 4, which is characterized by volcanic lithic sandstone and coal measures, containing distinct Late Triassic *Dicroidium* floras of inferred Carnian age (~230 Ma), dated by palynomorph biostratigraphy; these remains were deposited with sediments of high sinuosity rivers in a temperate climate with favorable seasonal growth^14^. The Fingal Valley deposits reveal stark links with palynological assemblages of the Carnian in Europe, i.e. coeval taxa in the Dolomites in Italy, which comprise spores of inferred hygrophytic environments (*Annulispora, Aulisporites*, and tricassate spores, likely *Camarozonosporites*). The resin that Fingal Valley amber represents was probably secreted by conifers (e.g., Cheirolepidiaceae), comparable to other Late Triassic deposits containing amber. The distinctive cheirolepidiacean palynomorph, *Classopollis*, shares an approximately coeval first appearance in southeastern Australia^15^. It is unlikely to be a coincidence that the oldest Mesozoic ambers are dated as Carnian in age, an interval represented by the ‘Carnian Pluvial Event’^16^, a time of major climatic shifts, seemingly worldwide, associated with major volcanism that saw increased rainfall during the late early Carnian and lower rainfall at the end of this age. It was a perfect climatic recipe for the secretion of resins and the associated sedimentary regimes of fluvial cycles that preserve amber in the geologic record^17,18^. Whilst most Carnian amber has been found in a narrow low northern paleolatitude band, the new Carnian-aged amber from Fingal Valley, Tasmania, provides tantalizing new evidence of Pangean-wide, increased resin production during the Late Triassic.

### Cretaceous amber

Significant amounts of early Late Cretaceous (late Cenomanian–Turonian, ~96–92 Ma) amber^19^ have been recently recovered in 2015–2016 from the Tupuangi Formation of Pitt Island, Chathams Islands, eastern Zealandia (Fig. 2D–F) (see also the Supplementary Text). In contrast to the small tubular ambers reported previously as *in situ* resin canals within fossils of cupressaceous conifers (Fig. 2F)^2^, these Chatham ambers represent relatively large droplets, blebs and fragmented pieces (≤2 cm in diameter) throughout over 300 m of section. The base of the Tupuangi Formation consists of a sandstone to granule conglomerate facies, but the majority of the strata (>300 m thick) comprises a facies association of fine- to medium-grained sandstone interbedded with carbonaceous siltstone facies. The latter facies association hosts abundant organically-preserved plant fossil remains, including rare horizons of amber. Multiple paleosols with densely-spaced tree trunks in growth positions were observed, and numerous horizons of well-preserved vegetative remains that were dominated by conifers (primarily Cupressaceae, Podocarpaceae and Araucariaceae), but locally abundant ginkgos, seed ferns, angiosperms and ferns, and rare lycophytes and bryophytes^20–24^. These strata represent deposition in fluviodeltaic alluvial and coastal plains with intermittent swamps, oxbow lakes and/or mires^19,20^. The fossils represent south-polar (~80–70°S)^25^ forests thriving during a global greenhouse interval, while still attached to the West Antarctic sector of Gondwana prior to Late Cretaceous continental break-up^26^. Significantly, these terrestrial ecosystems are the most southerly recorded Cretaceous forests and the most southern occurrence of Cretaceous amber. The potential of finding arthropods in the Tupuangi Formation amber is high, especially given a recent report^27^ that describes three species of beetles as compression fossils from these deposits.

Late Cretaceous amber pieces (N = 5) from the Santonian (~86–84 Ma) Tuna-1 petroleum well in the Gippsland Basin of southern Victoria (see also the Supplementary Text), bolster the Cretaceous Australian record, represented by a sole report^1^ on the Otway Basin Turonian amber. The Tuna-1 amber is dated by its occurrence in the Golden Beach subgroup of the Latrobe Group and spore-pollen in the *Tricolporites apoxyexinus* Zone^28^. The Tuna-1 amber is represented by a dominance of mostly transparent with rare semi-opaque orange and rare red amber that ranges from lenticular, angular, wedge-shaped, and irregularly shaped pieces. Possible filamentous inclusions have been noted in amber pieces obtained at 2668.1 m depth; otherwise, only minor amounts of pyrite, pseudoinclusions *sensu* Thiel *et al*.^29^ and particulate debris have been noted in the amber. The lowermost levels containing amber at 2673.1 m is characterized by siltstone with dispersed organic matter, which is overlain by sandy siltstone with coalified wood fragments, massive siltstones with coal laminations and organic matter, and capped by partially sideratized siltstone with poorly developed laminations and minor bioturbation.

### Paleogene amber

Early to mid-Paleocene (~66–62 Ma) amber has been recovered from the Department of Primary Industries (DPI) Core Lab in small amounts (N = 6) in 2013–2015 from drill cores in the Gippsland Basin of southern Victoria, Australia, in the petroleum well Barracouta-1 (spudded in December 1964; originally named the Gippsland Shelf-1 well) at 1967.9 m depth. This record is represented by translucent to semi-opaque orange and red amber devoid of inclusions to date, apart from particulate debris and pseudoinclusions^29^. This Paleocene amber from the lower *Lygistepollenites balmeii* spore-pollen Zone was deposited in sediments of the mid-Cretaceous to Eocene Latrobe Group, which mostly comprises fluvial, floodplain and coastal plain siliciclastics interspersed with thin coal beds^30^. Amber-bearing wells of the Gippsland Basin are located in the western portion of the Central Deep: an elongate, east-west striking depocenter that developed in response to rifting throughout the Late Cretaceous^31^. Bathymetry maps indicate that the amber-bearing wells only occur in the shallow portions of the Central Deep, with the most prolific deposits of the Tuna-1 well located along the shelf edge.

Early Eocene amber has been discovered recently in 2014 from the lower Paleogene Macquarie Harbour Formation of western Tasmania cropping out near and in the town of Strahan and along Macquarie Harbour (Figs. 2G–I and S1) (see also the Supplementary Text, discussing the primary collecting sites, including the Regatta Point Tavern and Railway Workshop areas). Our research indicates that this formation is much more extensive that previously reported and the base could well extend into the Paleocene. Entire droplets, runnels and variously shaped and sized fragmental pieces have been analyzed for inclusions. Most of the pieces are within the 1–2 mm range, but some exceed 20 mm. Palynological assessment has shown that the deposition of the resiniferous lignitic strata occurred during the Ypresian (middle to upper *Malvacipollis diversus* spore-pollen Zone; ~54–52 Ma)^32^, which marked a global and protracted greenhouse climate that saw a low to moderate thermal gradient from pole to equator^33^ and a possible increase in precipitation levels at high latitudes. Many ancestors of the modern tropical flora were able to disperse polewards and proliferate in areas that straddled the southern most regions of Gondwana. Strahan (42°S, 145°E), which is currently settled on the Macquarie Harbour, western Tasmania, was once situated at a latitude near the Antarctic Circle (66–64°S)^34^. During this time, the landscape was dominated by an interplay of channel and tidal systems, although the production, and consequent deposition, of resin potentially occurred within a freshwater swamp or estuary; this is evident from fine, highly carbonaceous silts and sand, the presence of sulphides that had subsequently oxidized, and the occurrence of mangrove *Nypa* and abundant dinoflagellates in the fossil record^3^. The early Eocene Tasmanian amber represents Class 1b ambers *sensu* Anderson *et al*.^35^. The botanic provenance of the amber is most likely to have been the conifer families Araucariaceae and/or Cupressaceae, as supported by FTIR and NMR analyses^36^, whilst macrofossils and pollen inclusions indicate the genera *Agathis* or *Araucaria*.

Abundant amber and associated diverse bioinclusions have been discovered in 2014 in the Anglesea Coal Measures near the town of Anglesea, Victoria, Australia. Late middle Eocene (~42–40 Ma; middle *Nothofagidites asperus* Zone)^37^ arthropods, plant matter, and fungal inclusions in amber have been identified from a single, continuous brown coal seam ~2 m thick directly overlying a paleosol with rootlets preserved (Figs. 2J–L and S1) (see also the Supplementary Text). The inclusions have been recovered from the ‘A Group Coal Seam’ in the middle–upper Eocene Eastern View Group, which comprises mostly non-marine claystone, sandstone and brown coal. The depositional environment of the Eastern View Group has been interpreted as a meandering river on a coastal plain with an indication of increasing marine influence up succession^38,39^. The preservation of the amber recovered from Anglesea varies from diagenetically altered pieces to well-preserved, vitreous examples with easily seen inclusions (Fig. 2K). The amber is mostly commonly light green or dark yellow and translucent with less common orange and red amber and rare white and brown amber. Most resin pieces are 1–2 mm long, but some exceed 40 mm with most being angular to subangular with fewer pieces being spherical and tabular in nature. Amber droplets are encountered within coal hand samples. Both Class Ib and Class II ambers^35^ were encountered at Anglesea. FTIR and NMR spectral data indicate that, as with the Tasmanian Macquarie Harbour Formation amber, Cupressaceae and/or Araucariaceae were primary botanical sources, but the Anglesea ambers also include one or more angiosperm groups with a chemical signature similar to Dipterocarpaceae^36^.

### Animal, plant and fungi inclusions in Paleogene amber

Diverse inclusions of arthropods, plants and fungi have been discovered in both the Macquarie Harbour Formation (MHF) of western Tasmania, dated as early Eocene (~54–52 Ma), and the ‘A Group Coal Seam’ of the Anglesea Coal Measures (ACM), which is late middle Eocene in age (~42–40 Ma) (see also the Supplementary Text).

**Figure 3:**
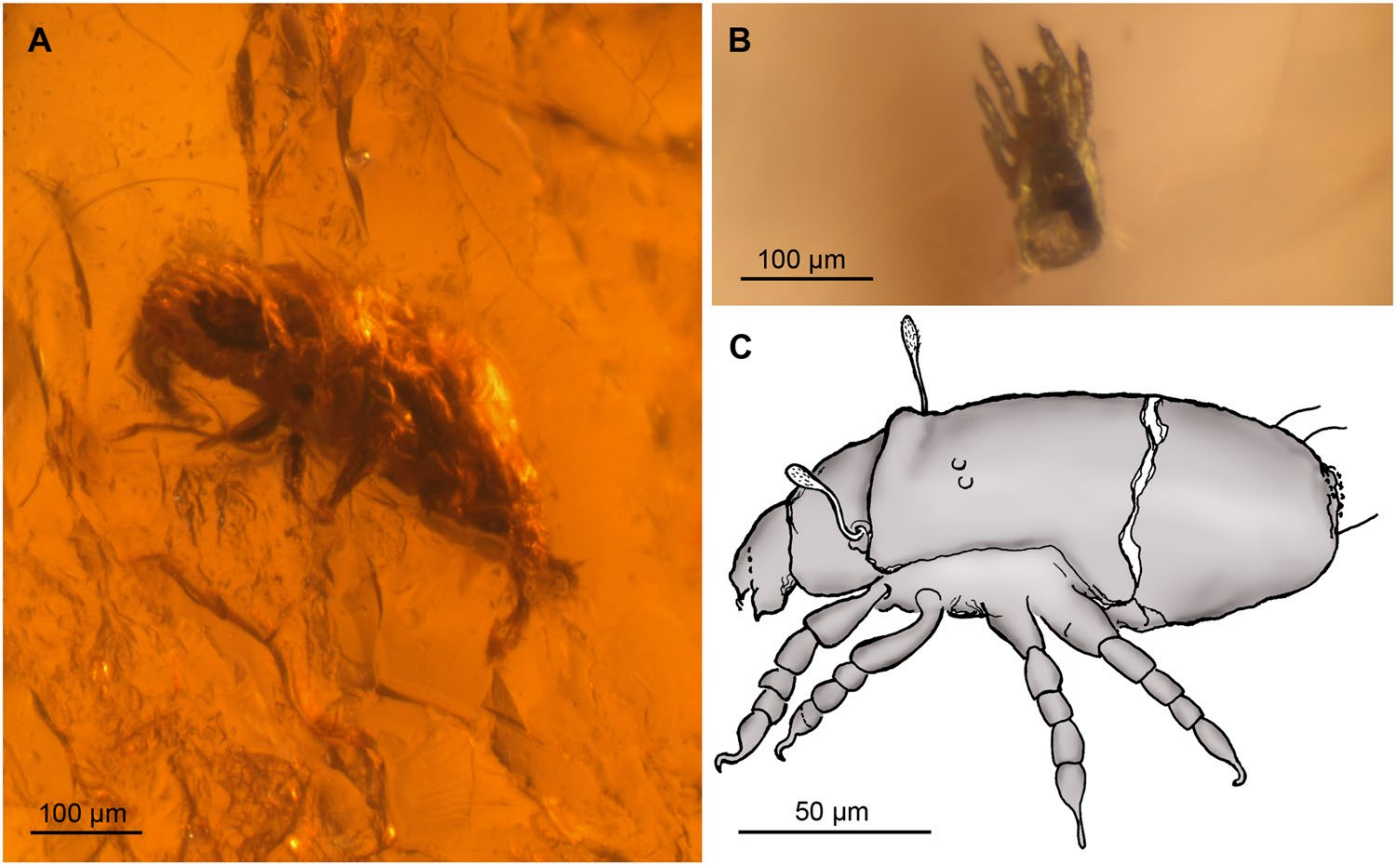
Arthropod bioinclusions in western Tasmania amber of Strahan, early Eocene. (**A**) Light photograph of a coccoidean 1^st^ instar (Hemiptera: Sternorryncha: Coccoidea), possibly of the family Eriococcidae. (**B** to **C**) Light photograph in dorsal view and camera lucida drawing (Adobe Photoshop CS2, version 9.0; www.adobe.com) in lateral view of an oribatid nymph, probably belonging to the Infraorder Enarthronota (Arachnida: Acari: Acariformes; most likely family Hypochthoniidae or Brachychthoniidae).

More than 2,530 amber pieces, representing a dominance of translucent pieces from deep red to clear, were assessed for bioinclusions in the MHF amber. Notably, two complete arthropods (an insect and a mite) have been identified along with an another decayed arthropod, possible evidence of termites, pieces of arthropods (femur, wing), coprolites, and nematodes; these nematodes (Fig. S1) are the oldest record of this group in the Southern Hemisphere, and the only previous occurrence being from the Miocene of New Zealand^9^. Palynological inclusions are observed, including an araucariacean pollen grain, along with fungal hyphae and probable fungal spores. Filamentous structures are detected in a number of specimens, as was disseminated organic material and potential plant structures, in addition to fungal mycelia. Many spherical inclusions of varying sizes are common with a high proportion being attributed to a different plant exudation that did not mix with the coeval resin exudation (named as pseudoinclusions by other authors). The complete insect is a coccoidean 1^st^ instar, possibly of the family Eriococcidae (Fig. 3A), thus representing a major group of scale insects with a fossil record extending back into the mid-Cretaceous of Australasia^40^. The mite (Fig. 3B,C) is an oribatid nymph and probably belongs to the Enarthronota (most likely Hypochthoniidae or Brachychthoniidae, being both cosmopolitan edaphic families).

**Figure 4:**
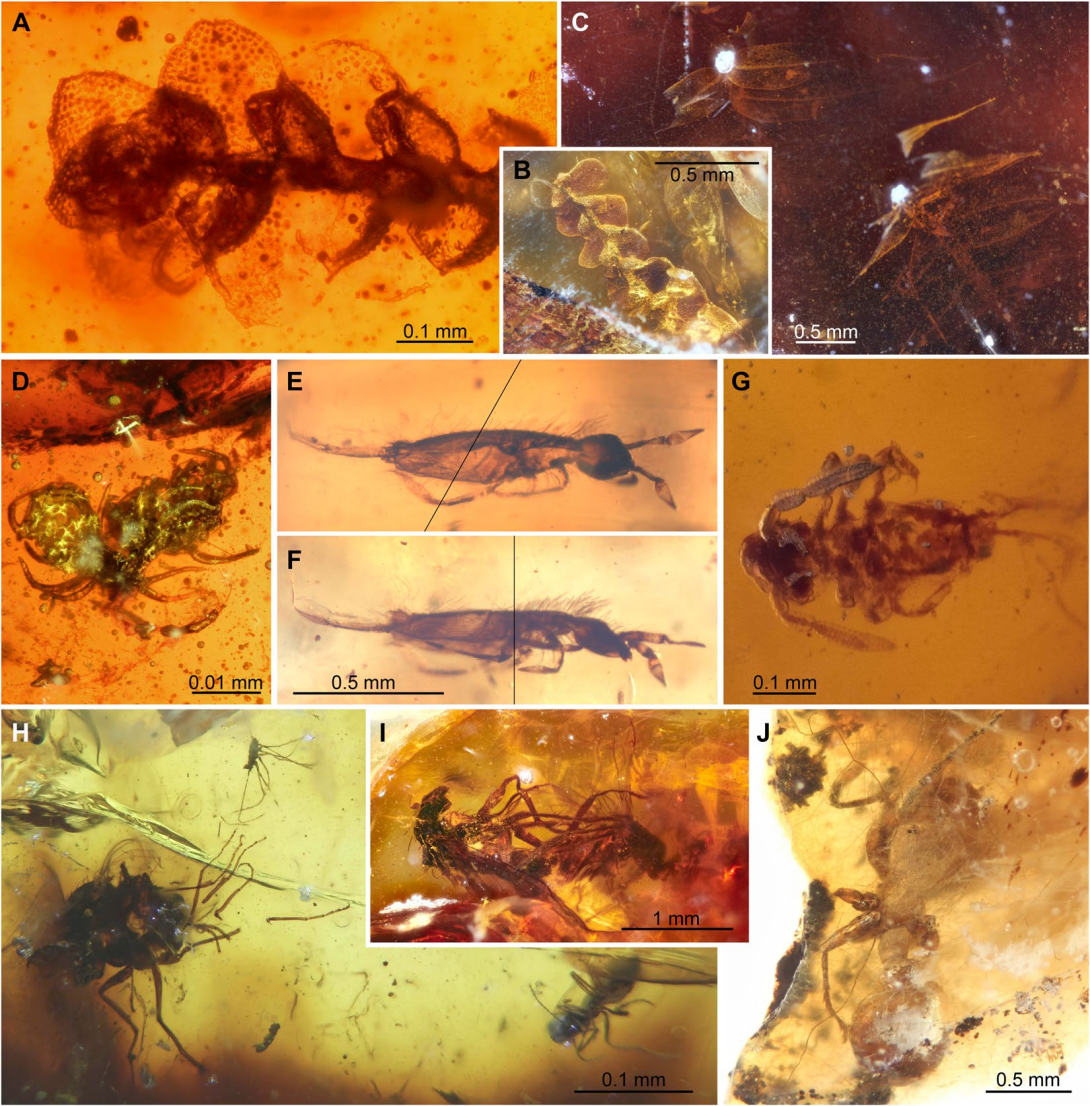
Significant bioinclusions of plants and animals in Southern Gondwana late middle Eocene amber of Anglesea, Victoria. (**A** to **B**) Liverworts of the genus *Radula* (Marchantiophyta: Radulaceae). (**C**) Two stems with perfectly preserved phyllids or leaf-like structures of mosses of the genus *Racopilum* (Bryophyta: Racopilaceae). (**D**) Juvenile individuals of spiders. (**E** to **F**) Springtail of the living genus *Coecobrya* (Entomobryomorpha: Entomobryidae) in two views. (**G**) A Symphypleona springtail. (**H**) Light photograph of large piece of yellow amber with two dipterans, Dolichopodidae at left and Ceratopogonidae at right, and at top of image a mite of the living genus *Leptus* (Arachnida: Acari: Trombidiformes: Erythraeidae). (**I**) Dipterans of the family Dolichopodidae (long-legged flies) in copula. (**J**) Worker ant of the living genus *Monomorium* or a “*Monomorium*-like” lineage (Hymenoptera: Formicoidea: Formicidae) (see Fig. S2).

**Figure 5:**
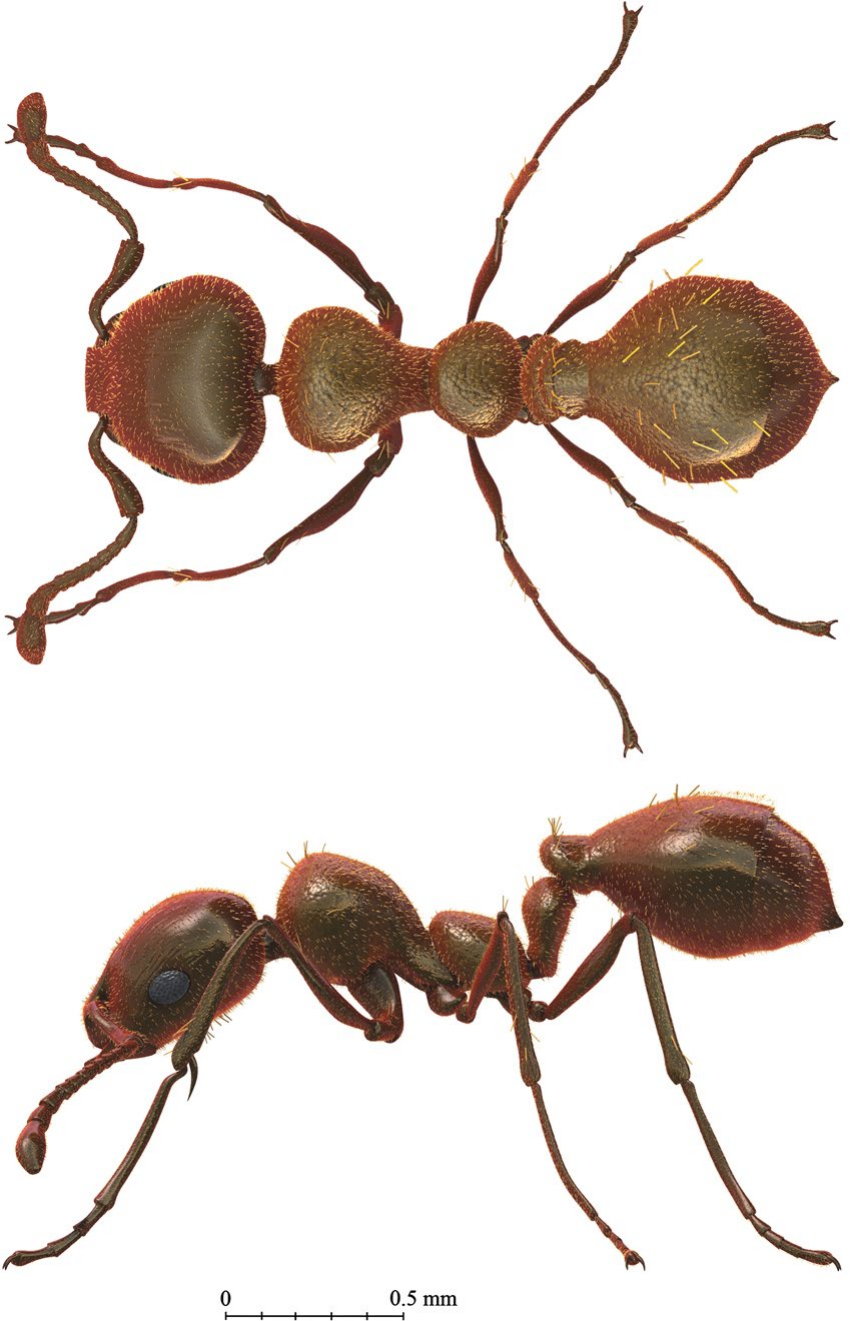
Reconstruction with anatomical features of the second fossil ant discovered in Australasia and the oldest from Southern Gondwana. The reconstruction of this worker ant corresponds to a new myrmicine species belonging to the extant genus *Monomorium* or a “*Monomorium*-like” lineage discovered in late middle Eocene amber of Anglesea, Victoria (see Fig. 4J). It is based on several specimens (also see Fig. S2), and the body color pattern is conjectural, but aligns with the common pattern found in extant *Monomorium* ants. Performed using Light-Wave 3D computer graphics program (NewTek; www.newtek.com/lightwave/) (Artist: J.A. Peñas).

**Figure 6:**
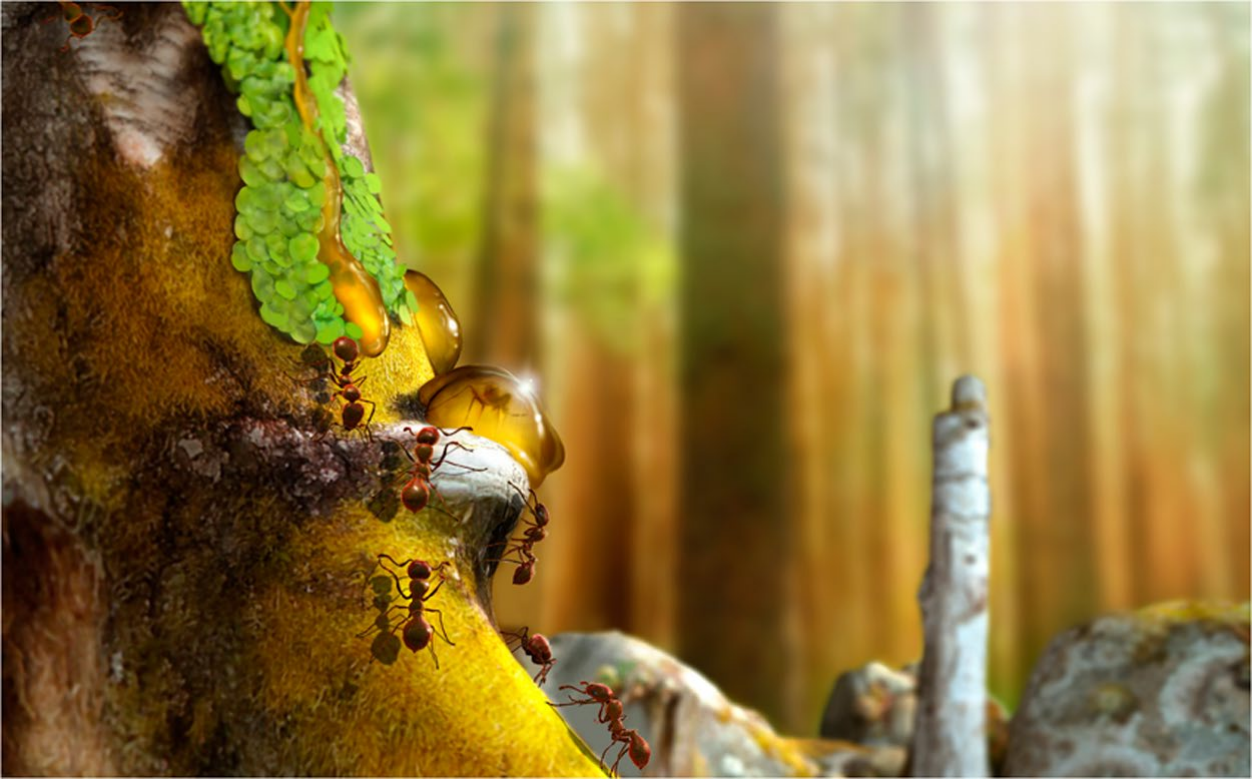
Paleobiological reconstruction of the new “*Monomorium*” ant and the liverwort *Radula* sp. The ants and liverwort are represented on a resiniferous conifer tree trunk, and this scenario is reconstructed from the assemblage preserved in late middle Eocene amber of Anglesea, Victoria. Performed using Light-Wave 3D computer graphics program (NewTek; www.newtek.com/lightwave/) (Artist: J.A. Peñas).

The ACM amber biota comprises a multitude of animal, plant and fungal groups from a total of 3,291 amber pieces and the most bioinclusion-rich, *in situ* deposit recorded from Australia (Fig. 4). Fungi include hyphomycetean remains similar to the extant anamorphic genus *Monotosporella* (Fig. S1). Plant inclusions comprise well preserved non-vascular plants (i.e., bryophytes) of liverworts and mosses (the first in amber in the far south), a trichome, a petal, leaf bract, possible seeds, and a possible megaspore. Significantly, two species of the liverwort *Radula* (Radulaceae) have been recognized along with two moss species attributed to *Racopilum* (Racopilaceae) (Fig. 4A–C). Several arthropods (arachnids and hexapods) have been identified in the ACM amber, which include juvenile spiders in a cluster most likely formed after hatching for safety reasons until the next moult (Fig. 4D) and immature mites belonging to the Trombidiformes (Erythraeidae, *Leptus* Latreille, 1796). Hexapods are represented by some collembolans and diverse insect groups, as lepidopterans represented by diverse and abundant, isolated scales (Fig. S1), and flies of the families, Ceratopogonidae (or biting midges; Fig. 4H), Dolichopodidae (or long-legged flies, two of them in copula; Fig. 4I), and Tipulidae (or craneflies). Dolichopodidae have a fossil record extending to the Cretaceous with several records in the Paleogene in amber deposits worldwide. These predatory flies today eat springtails (also recorded in the ACM amber), aphids and a variety of larvae in a wide range of habitats near water or in meadows and woodland edges. Some are restricted to wet places including sands on the banks of water bodies, including saline water and also the intertidal zone adjacent to seashores. Significantly, the mating flies represent an extremely rare example of frozen behavior in the Australian fossil record. Ceratopogonidae is a globally distributed family. Its fossil record extends back into the Jurassic. Adults feed on nectar, but many females are haematophagous (blood suckers). Larvae live usually in moist environments. In Australia, the oldest record of this family is a poorly preserved compression fossil in the Early Cretaceous Koonwarra beds of Victoria, most likely belonging to the pantropical and relict genus *Leptoconops* Skuse, 1889^41^. In our amber, the family is represented by two complete specimens, a male and a female, plus an incomplete specimen, all present in the same small portion of amber, representing the cosmopolitan genus *Culicoides* Latreille, 1809. More significant is a complete female specimen of the genus *Meunierohelea* Szadziewski, 1988 (Fig. S1), which persists in the Recent fauna with one extant species in Australia while its past distribution was wider, including Baltic, Bitterfeld, Rovno and Indian Cambay ambers^42^. Some other ceratopogonid relict genera are found as fossils in the Northern Hemisphere, revealing limited modern distributions in the Southern Hemisphere as *Austroconops* Wirth and Lee, 1958 (Australia), *Metahelea* Edwards, 1929 (Philippines and Australia) and *Physohelea* Grogan and Wirth, 1979 (Patagonia)^43^. Borkent and Craig^44^ proposed one hypothesis in which competition with Ceratopogonini (especially *Culicoides*) have replaced *Austroconops* from most of its historical range explaining this type of present distribution. Other degraded and partial insects include several other dipterans, a probable member of Tingidae or ‘lace bugs’ and a confirmed cockroach (Blattodea). Two collembolans belong to the so-called ‘slender springtails’ (Hexapoda: Entomobryidae) that, until now, had no fossil record in Australia, but are found elsewhere in the Eocene, including many specimens in Baltic amber. One of these specimens has been classified in the living genus *Coecobrya* (Fig. 4E,F). The fossil record of springtails is notably poor in the Southern Hemisphere, but Entomobryidae have already been recorded as amber inclusions from the late Oligocene–early Miocene of New Zealand^9^. Exquisitely preserved specimens of collembolans belonging to Symphypleona have been also recovered from the new ACM amber locality (Fig. 4G). Most significant are several winged and worker ants belonging to the myrmicine genus *Monomorium*, according to the current status of this non-monophyletic genus (they are very similar to the synonymized *Chelaner*), or a “*Monomorium*-like” lineage. The specimens exhibit a unique combination of characters indicating that most likely this is the fourth known fossil species of *Monomorium* (Figs. 4J, 5, 6 and S2) and the first recorded in the Southern Hemisphere^45^. In the Recent biota, *Monomorium* mostly inhabits the Old World, particularly the tropics, and Australia is considered one of the two main centers of speciation. These new fossil ants are the oldest from Southern Gondwana and unveil the antiquity of many elements in the modern Australian biotas.

## Discussion

The number of amber occurrences in the Southern Pangea and Southern Gondwana regions has been boosted by recent discoveries over the last five years of inclusion and fossil-rich deposits dating from the Late Triassic to early Paleogene of Australia and eastern Zealandia.

These occurrences are an advance in amber paleontology, providing us with significant, new paleobiogeographic data. Upper Triassic terrestrial deposits of the Fingal Valley Coal Measures provide a tantalizing new record of early Mesozoic resin produced near the polar circle (~65–70°S) in the Southern Hemisphere with the potential to recover more material within the widespread terrestrial deposits of Tasmania. The newly discovered early Late Cretaceous amber of southeastern Australia and the Chatham Islands (New Zealand) originated in south-polar to subpolar forests during the mid-Cretaceous ‘hothouse’. Paleocene amber from Victoria is the first record of this age in the Southern Hemisphere. The lower Eocene Macquarie Harbour Formation and upper middle Eocene Anglesea Coal Measure amber biotas provide the oldest recorded fungal, plant and animal bioinclusions from Southern Gondwana. These bioinclusions exhibit the exceptional preservation of the finest of external anatomical details allowing complete descriptions of new taxa. They not only provide taxonomic and phylogenetic data, such as new species and the oldest fossil ant discovered from Southern Gondwana, but also include rare “frozen behaviors”, such as the mating of two flies, and representatives of diverse trophic levels in the ancient resiniferous forests as predators such as spiders, detritivores such as springtails, and phytophages such as scale insects. Despite concerted efforts by many researchers for well over a century, except for the Oligocene-Miocene ambers of Southern New Zealand, no early Mesozoic or pre-Neogene amber with animal and plant inclusions, has been recovered from Southern Pangea and Southern Gondwana until this report with scores of new records and vast potential for future finds.

## Materials and Methods

### Amber preparation

As much of the amber was concealed within an organic matrix, the physical extraction of specific amber fragments was conducted considering the fragility of many of the pieces, which fracture quite readily. In order to ensure optimal preservation of potential inclusions, the bulk samples, comprising both amber and matrix, were soaked in water between two and seven days. As the surrounding rock became malleable with saturation, the samples were manually broken by hand into smaller pieces, and any recognized part and counter-part portions were gently separated from the matrix with a dissecting needle, and placed together in separate vials of tap water and labeled accordingly, to represent immediate sample relation.

The remainder of the soaked sedimentary samples were gradually worked through a set of four stainless steel sieves. These Endecotts laboratory test sieves had calibrated apertures to the order of 4 mm, 2.8 mm, 2 mm, and 1 mm, and enabled the removal of the fine organic sediments, while categorizing the substantial amber fragments into different sizes. Amber segments less than 1 mm were present in the bulk samples, but were excluded in the current study due to time constraints on processing and analysis. Once more, any fossil resin piece that was seen to fracture during the sieving process was removed with forceps and categorized in a separate vial, while the remaining liberated amber was placed in collective vials differentiated by size and location (e.g., “RWW > 1 mm”, “Sub-CableB > 3 mm”).

Following the primary extraction of amber from each bulk sample, the fossil resin needed to be prepared for viewing. Although much of the fragmented amber was exceptionally clear, many of the whole-resin droplets comprised a sugary-rind, likely caused by oxidative weathering that enveloped the pristine glass within. To remove this surface, each specimen was clasped by forceps and viewed under a microscope, while submerged in water, as a scalpel was used to scrape away the opaque surface. The removal of said surface contaminants allowed for the observation of the amber under transmitted light microscope, and thus, to recognize potential inclusions. As inclusions were identified, the relevant fossil resins were separated into individual vials and catalogued. Latex gloves were worn at all times during the handling of these samples to ensure biogenic oils from the processer did not contaminate the amber for future geochemical analysis. Particularly significant amber pieces with bioinclusions were embedded in Epotek 301 epoxy resin and in some case further cut and polished with cesium oxide for a final mirror-like finish to view the inclusion, per highly successful outcomes performed at the Instituto Geológico y Minero de España (IGME), Madrid.

Amber samples from the Late Triassic and early Paleocene cores were treated differently for microscopic assessment. Amber was collected from the cores either manually using forceps or by cutting a small piece from the core such that the amber remained embedded in matrix. Excess matrix was removed using a rock saw, and the amber was cleaned and polished using wet silicon carbide abrasive paper at successively finer grit (FEPA P 600–3000 or 7–25.8 μm) to expose a viewing surface. Like many Cretaceous ambers, the Otway Basin amber is brittle and many samples were either collected as broken fragments from the core or burst during extraction. In order to preserve large pieces intact and aid in visual inspection, select samples were cleaned and embedded in Epotek epoxy resin following the method of Nascimbene and Silverstein^46^. Each whole piece and fragment of burst amber was examined using transmitted light under a Leica M80 and also a Leica DM2500P microscope fitted with a Leica DFC 290 HD camera and photographed using the Leica Application Suite (LAS) software, version 3.8 and the Montage Multifocus module. In cases where multiple amber pieces were contained within a single rock sample, some of the amber was left embedded in matrix for preservation. Permission to collect samples from core was granted by the Department of State Development, Business and Innovation (State Government, Victoria), the Department of Infrastructure, Energy and Resources (Tasmanian Government), and by Weatherford Laboratories (Brisbane, Australia).

Chatham Islands specimens are housed at GNS Science, Lower Hutt, New Zealand, and assigned unique sample registration numbers, see^2^. Individual specimens recovered from the same bulk sample were given letter specimen codes. Ambers associated with plant fossil samples have prefix ‘PL’, and ambers from bulk sediment samples have prefix ‘B’. Bioinclusions are housed at the Museums Victoria (Australia) paleontology collection.

### Imaging

Line drawing was prepared using an Olympus U-DA drawing tube attached to the Olympus BX51 compound microscope and by using Adobe Photoshop software (CS2, version 9.0; www.adobe.com). The map was generated using Adobe Illustrator CC2018 (www.adobe.com) and derived from sketching the region from Google Earth (www.earth.google.com). Micrographs were taken using a both ColorView IIIu Soft Imaging System attached to an Olympus BX51 compound microscope at the IGME (Madrid) and a Vision Dynamic BK Lab Imaging System in the School of Earth, Atmosphere and Environment at Monash University (Melbourne, Australia). Anatomical ant reconstruction and paleobiological reconstruction (Figs. 5, 6) were performed using the Light-Wave 3D computer graphics program (NewTek; www.newtek.com/lightwave/).

See the Extended Materials and Methods in the Supplementary Material for more details on amber sites and on geologic setting and age dating of fossiliferous Eocene amber.

## Data Availability

All data needed to evaluate the conclusions in the paper are present in the paper and/or the Supplementary Materials. Correspondence and material related to this paper may be requested from Jeffrey D. Stilwell (Jeffrey.Stilwell@monash.edu) and Enrique Peñalver (e.penalver@igme.es).

## Supporting Information

Supporting Information.
