# Supplementary material for: Amber from the Triassic to Paleogene of Australia and New Zealand as exceptional preservation of poorly known terrestrial ecosystems
Source: Sci Rep. 2020 Apr 2;10:5703. doi: 10.1038/s41598-020-62252-z (PMC7118147; doi:10.1038/s41598-020-62252-z)
Supplement: Supplementary file 1 — Supporting Information. [file 41598_2020_62252_MOESM1_ESM.docx]

**Supplementary Information**

**Amber from the Triassic to Paleogene of Australia and New Zealand as exceptional preservation of poorly known terrestrial ecosystems**

Jeffrey D. Stilwell^*^, Andrew Langendam, Chris Mays, Lachlan J.M. Sutherland, Antonio Arillo, Daniel J. Bickel, William T. De Silva, Adele H. Pentland, Guido Roghi, Gregory D. Price, David J. Cantrill, Annie Quinney and Enrique Peñalver^*^

^*^Corresponding authors. Emails: Jeffrey.Stilwell@monash.edu (J.S.); e.penalver@igme.es (E.P.)

**Extended Materials and Methods**

Site Descriptions

*Late Triassic*

Triassic amber of Carnian age was discovered on the 13^th^ and 14^th^ March 2015 in the Fingal Valley Coal Mine of north-eastern Tasmania from cores housed at the Department of Mines (Hobart, Tasmania), specifically from Valley Mine No. 2 at S41° 38.037’ and E148° 03.203’, project F/FV2—EL16/2010, Fingal, VR002—‘A’ Seam, Part 2, logged originally by W. Hagell on the 19^th^ January 2012, but there was no previous mention of amber present. Sample FV2 from VR2 #12 contained small <1 mm pieces of crystalline, clear amber at the 44.9 m marker. Sample FV3 from VR2 #22 also contained small amber clasts of a predominantly opaque nature in a thick conglomeratic layer between 83.0 to 79.32 m depth. One small fragment of amber was found in FV4 from VR2 #28 at 102.0 m depth and small pieces were noted also in FV5 from VR2 #27 scarcely below 101.0 m depth.

*Late Cretaceous (Cenomanian, Chatham Islands)*

Amber has been recovered from 21 samples of the Chatham Islands. All but one of these localities are from strata of Waihere Bay, northwest Pitt Island (locality numbers: CH/f 0784, CH/f0799, CH/f0807, CH/f0814, CH/f0816–0828). One additional locality is from southern Tupuangi Beach, northeastern Pitt Island (CH/f0815). All samples are housed at GNS Science, Lower Hutt, New Zealand (*48*). Additional stratigraphic details in (*2*).

*Late Cretaceous (Santonian*–*middle Campanian) and early Paleocene (Gippsland Basin)*

The Santonian–middle Campanian amber has been recovered from samples ranging from 2673.10 to 2662.10 m from core samples only from Tuna-1. The early Paleocene amber is represented by fragments in core samples from the Barracouta-1 well only (lat. S38°16.683’, long. E147° 42.750’) only at a depth of 1968.10 m, where it is positioned in southeastern Australia approximately 200 km east of the city of Melbourne. Cores are stored at the Geological Survey of Victoria’s Drill Core Library, Werribee, Victoria (Australia); amber samples are housed at the Melbourne Museum, Museums Victoria (MV), Australia.

*Early Eocene*

Initially only one lignitic exposure, at the Railway Workshop Outcrop (termed Regatta Point in (*3*)), was seen to possess traces of fossil resin. However, upon closer inspection, a small amount of transparent amber was also present at Regatta Point Tavern. Extensive surveying of the eastern coast of Lettes Bay, which included Risby Cove and the Regatta Point Peninsula, led to the recognition of four more locations that accommodated amber-rich lignitic layers. The major deposit of amber was coined Submarine Cable (SubCable), while three minor subsidiaries identified along the coast to the south were designated Submarine South 1, 2 and 3 (SubSth-1, -2, and -3). All Macquarie Harbour Formation samples are housed at the Melbourne Museum, Museums Victoria (MV), Australia, and provided with museum registration numbers (prefix “P”).

Regatta Point Tavern (RPT) – S42°09.723’ / E145°19.579’

The Regatta Point Tavern consisted of a 10 m outcrop, in which the lignitic layer is represented at the 1.5 m mark. Only minute traces of transparent amber are recognizable, with remnants of fossil resin in the form of large completely oxidized structures prevalent throughout the strata. The sediments of the upper *M. diversus* Zone are unconformably overlain by an interbedded Pleistocene sandstone-polymict conglomerate system, which consists of sandstone intraclasts, metamorphosed sedimentary rocks (i.e., slates), vein quartz, and porphyritic volcanic pebbles.

Railway Workshop (RWW) – S42°09.727’ / E145°19.676’

The RWW outcrop consists of two segments that are dissected by a railway line. The lower segment comprises a meter of carbonaceous light brown silts to fine sands, which encompasses a vast amount of leaf macrofossils. The resiniferous lignitic stratum, with a strike /dip reading of 148°/15°E, was located within the upper segment at approximately three meters from the base. This site produced a high yield of transparent fossil resin with a greatly reduced ratio of diagenetically weathered amber. As is apparent at RPT, the top strata of the Ypresian sediments have been unconformably superimposed by a cobble to boulder sized polymict conglomerate. It should also be noted that there was a high degree of sulfur deposition throughout the outcrop, particularly in the lower carbonaceous silts and the amber-rich lignite.

Submarine Cable (SubCab) – S42°09.988’ / E145°19.751’

The Submarine Cable deposit is located almost directly on the coast within a small, three-meter exposure that is overlain with Pleistocene cobble gravel. On first appearances, there appears to be two lignitic layers that run parallel to one another. The upper layer (SubCab-B) strikes N140° and dips 70° to the east. The lower stratum (SubCab-A) expresses a similar bedding attitude; however, upon investigation it was determined that the layer only extended 30 cm below the surface, and so was defined as an erosional slump that has stemmed from SubCab-B. Both SubCab-A and -B possessed fossil resin, although SubCab-B was far more prolific, being one of the most abundant sources of amber in the study.

Submarine South 1 (SubSth-1) – S42°10.078’ / E145°19.776’

Submarine South 2 (SubSth-2) – S42°10.064’ / E145°19.775’

Submarine South 3 (SubSth-3) – S42°10.047’ / E145°19.767’

Each of the three Submarine South deposits consisted of small lignitic exposures that were surrounded by loose Pleistocene-age cobble gravel, which has most likely been transported from inland through the erosion of the coastal geology.

*Late middle Eocene*

Five exposures were initially identified which contained fossil resins at the Anglesea Coal Measures, Alcoa Mine; however, subsequent stratigraphic correlation indicated that each sampling site represented coeval points along a single, continuous coal horizon. Whilst amber was observed at several different locations along the coal seam, the exposure identified in 2014 has been deemed the most prospective, and is the sole focus of the present study as a consequence. The middle to upper Eocene brown coal measures contained varying amounts of fossil resins across the mine exposure, resulting in the optical analysis of the 3,289 pieces of amber housed at Monash University, Victoria (Australia). All key bioinclusions will be housed in paleontology collection at Museums Victoria, Melbourne, Australia.

### “Unnamed Locality” (UL) – S38°23.491’ / E144°09.522’

The sample site UL is represented by ~2m of vertical outcrop, consisting of fine- to coarse-grained sandstone with lignite fragments and a brown coal seam represented at the 1.2m mark. The sandstone facies consist of moderate- to poorly-sorted and moderately well-sorted angular quartz grains with some cross-bedding. Within these facies, some quartz sand grains appeared subrounded to rounded. In strata directly overlying amber-rich coal seams, there was a high proportion of angular quartz sand grains. The sedimentary rocks are unconformably overlain by a series of upward-lightening coal lithotype cycles. Coal lithotype cycles are characterized by coal seams and intermittent clay successions with carbon content highest at the base and decreasing upsequence. Fossil resins were most abundant in coal seams directly overlying paleosols preserved with rootlets and were not found in the underlying strata or in association with leaf litter horizons.

Discussion about the autochthony and allochthony of Cape York amber

Cape York amber occurs only along a 120 km length of remote Coral Sea beaches between Cape Grenville and Cape Weymouth, northern Australia. Although the geological source and age of this amber is uncertain, hundreds of pieces have been gathered, ranging in size from a few millimetres to almost 200 mm in diameter and in a range of colours (*55*). Most pieces have been found washed up at the high tide strandline, although small amber pieces have been found *in situ* with lignite deposits behind foredunes on one Cape York beach. Both Cape York and Baltic ambers are distinguished by having a major occurrence as flotsam washed up on beaches.

Geologically, the area is dominated by Paleogene sediments which include the coastal lignites. The region is poorly known due to inaccessibility, the lack of outcrops and extensive cover by vegetation and Quaternary dune fields. Possibly, lignites eroded by monsoonal drainage systems are the source of the amber pieces, which are carried to the sea during the rains and redistributed along the coast by currents.

Colchester *et al*. (*56*) found the chemical composition of Cape York amber to be broadly similar to New Zealand kauri copal (i.e., *Agathis* sp.) but with a distinctive chemical signature. However, Sonibare *et al.* (2014) argued that the signature of the Cape York amber is that of Dipterocarpaceae, a major predominately Oriental tree family with greatest richness in Borneo and extending to New Guinea, but unknown from Recent Australia. They, therefore, contended that Cape York amber is not of local origin, but was probably transported by currents from a source in New Guinea or Southeast Asia. No amber yet has been found in New Guinea, but it is known from Brunei (Borneo). However, several points must be raised: i) Cape York amber only occurs along a highly restricted section of coastline, so if it had been dispersed from New Guinea or particularly southeast Asia, it certainly should be more widely distributed in Australasia; ii) some ambers are denser than sea water and would only survive long transport by turbulent coastal currents or wave action (as along the coast of the shallow Baltic Sea), but are unlikely to be carried across open ocean, where amber would soon sink to the sea bottom; iii) Cape York amber has been found *in situ* with coastal lignite deposits (55), suggesting a parautochthonous occurrence (*57*); iv) How diagnostic is the chemical signature for the family Dipterocarpaceae, and could analogues from other angiosperm families appear similar? Indeed, as cited in this paper, Coward *et al*. (*36*) suggest that in addition to gymnosperm sources, Eocene Anglesea amber from Victoria may have one or more angiosperm sources, of Class II amber (which includes the Dipterocarpaceae). Therefore, an alternative interpretation for the Class II amber at both Anglesea and Cape York is a native source from a presently unknown Australian angiosperm.

Further research to clarify the origin of the Cape York amber-bearing deposit is important considering that it is highly fossiliferous, and some 300 known inclusions include many insect families, spiders, mites, millipedes, a pseudoscorpion, and plant matter (flowers, pollen, leaves, seeds) and strands of mammalian hair. Bickel (*58*) described a fly in the genus *Chaetogonopteron* (Diptera: Dolichopodidae) from this amber. *Chaetogonopteron* is a rich extant Old World genus, which also occurs in northern Australia. The genus probably arrived in Australia by dispersal from the north as the Australian Plate drifted towards lower latitudes during the Paleogene, suggesting a maximum early Paleogene age for the amber.

Geologic Setting and Age Dating of fossiliferous Eocene amber

*Early Eocene of Western Tasmania, Australia*

The Macquarie Harbour Formation is located in southwestern Tasmania and is dominated by exposed lower Paleogene marginal marine strata. The Macquarie Basin itself is thought to have originated from rifting of the Paleozoic basement during the Cretaceous to early Paleocene. With the development of the Macquarie Harbour Graben, a thick sequence of Paleogene sediments accumulated, and has been contained within the bounds of a major fault along the eastern margin. The lithology of these strata consists of a mixture of defined interbedded sands, pebble to cobble-sized sediments, silts and clays, as well as siliceous Paleozoic Owen Group-derived clasts (*49*).

The age of amber-rich localities in the area (RPT, RWW, SubCab, SubSth-1, -2, -3) have been constrained through spore-pollen biostratigraphy to the upper *Malvacipollis diversus* Zone, potentially extending to the middle *M*. *diversus* Zone. The abundance of the fossil pollen *Spinisonocolpites prominatus* is critical as there is a distinct acme of this palm mangrove species (*Nypa*) in the upper *M. diversus* Zone, as compared to the successive *Proteacidites asperopolus* Zone (*50*). The second definitive piece of age dating refers to palynological data collected by Cookson and Eisenack (*51*). The study demonstrated the presence of the dinocyst *Wilsonidium ornatum*, in what is referred to as the *W. ornatum* microplankton Zone, a correlative to the upper *M. diversus* Zone (*51*). The evidence produced from these comprehensive studies places the relevant strata in the age bracket of 54–49 Ma.

The organic-rich lithofacies also incorporates an abundance of macrofossils, such as foliar compressions and well-preserved cuticles of plant material (*3*). Advanced mapping and analytical techniques have been used to investigate extensive faults, thrusts, and shear zones, and as a result, six incidences of folding have been recorded at multiple localities, indicating a series of complex deformation events since the deposition of the Macquarie Harbour Formation in the Eocene (*49*).

*Late middle Eocene of Victoria, Australia*

The Torquay Sub-basin is a small depression within the Otway Basin and primarily composed of the non-marine Paleocene–Eocene sediments of the Eastern View Group, which unconformably overlie the Lower Cretaceous Otway Group (*52*). The Eastern View Coal Measures was first defined with an interval of Boonah Sandstone at the top. These successions were later combined (*59*) to form the Eastern View Formation. The stratigraphic unit was given group status (*38*). The Boonah Sandstone was then redefined as the lower unit of the Demons Bluff Group therein, based on seismic mapping, which indicated a major regional unconformity at its base (*39*).

The middle–upper Eocene Eastern View Group is primarily composed of non-marine claystone, sandstone and brown coal facies. In its offshore component, the Eastern View Group is over 800 m thick in the Nerita-1 well (*39*). The majority of brown coal seams occur within the upper parts of the Eastern View Group and were successfully subdivided into groups C, B and A (*53*), which are found in the upper part of the succession. The Eastern View Group, Salt Creek and Anglesea formations were correlated (*54*) to the middle to upper Eocene “*Nothofagidites asperus* Biozone” (47–38.5 Ma), using the biostratigraphic scheme of Stover & Partridge (*50*).

**References**

48. Mays, C., Cantrill, D. J. & Bevitt, J. J. Polar wildfires and conifer serotiny during the Cretaceous global hothouse. *Geology* **45**, 1119–1122 (2017).

49. Quilty, P. G. *et al*., Cretaceous-Neogene evolution of Tasmania. In *Geological Evolution of Tasmania*, K. D. Corbett, P. G. Quilty & C. R. Calver, Eds., pp. 409–509. (Geological Society of Australia, Special Publication 24. Tasmania Division, 2014).

50. Stover, L. E. & Partridge, A. D. Tertiary and Late Cretaceous spores and pollen from the Gippsland Basin, southeastern Australia. *Proc. Roy. Soc. Vic*. **85**, 237–286 (1973).

51. Cookson, I. C. & Eisenack, A. Some Early Tertiary microplankton and pollen grains from a deposit near Strahan, western Tasmania. *Proc. Roy. Soc. Vic*. **80**, 131–140 (1967).

52. C. Abele *et al*. Chapter 8. Tertiary. In *Geology of Victoria*, J. G. Douglas & J. A. Ferguson, Eds., pp. 252–350. (Victorian Division, GSA, 1988).

53. George, A. M. The geology of the Anglesea Coalfield. State Electricity Commission of Victoria, Geological Report of the Coal Production Branch (unpubl. 1962).

54. D. Stanley, A preliminary appraisal of the groundwater resource potential of the Torquay Basin for urban supply development. HydroTechnology P/L Report cc/30400.001A/1 (unpubl. 1994).

55. Hand, S. *et al.* Australian Cape York amber. In B*iodiversity of Fossils in Amber from the Major World Deposits*, D., Penney, Ed., pp. 69–79 (Siri Scientific Press, Manchester, 2010).

56. Colchester, D. M., Webb, G. & Emseis, P. Amber-like fossil resin from north Queensland. *Aust. Gemmol*. **22**, 378–385 (2006).

57. Seyfullah, L. J. *et al*. Production and preservation of resins – past and present. *Biol. Rev*. **93**(3), 1684–1714, https://doi.org/10.1111/ brv.12414 (2018).

58. Bickel, D. J. The first species described from Cape York amber, Australia: *Chaetogonopteron bethnorrisae* n. sp. (Diptera: Dolichopodidae). *Denisia* **26**, 35–39 (2009).

59. C. Abele, *Anglesea 1:63 360* *Geological Map*. Mines Department Victoria, Melbourne (1968).

60. Sadowski, E. M. *et al*. The anamorphic genus Monotosporella (Ascomycota) from Eocene amber and from modern *Agathis* resin. *Fungal Biol*. **116**, 1099–1110 (2012).


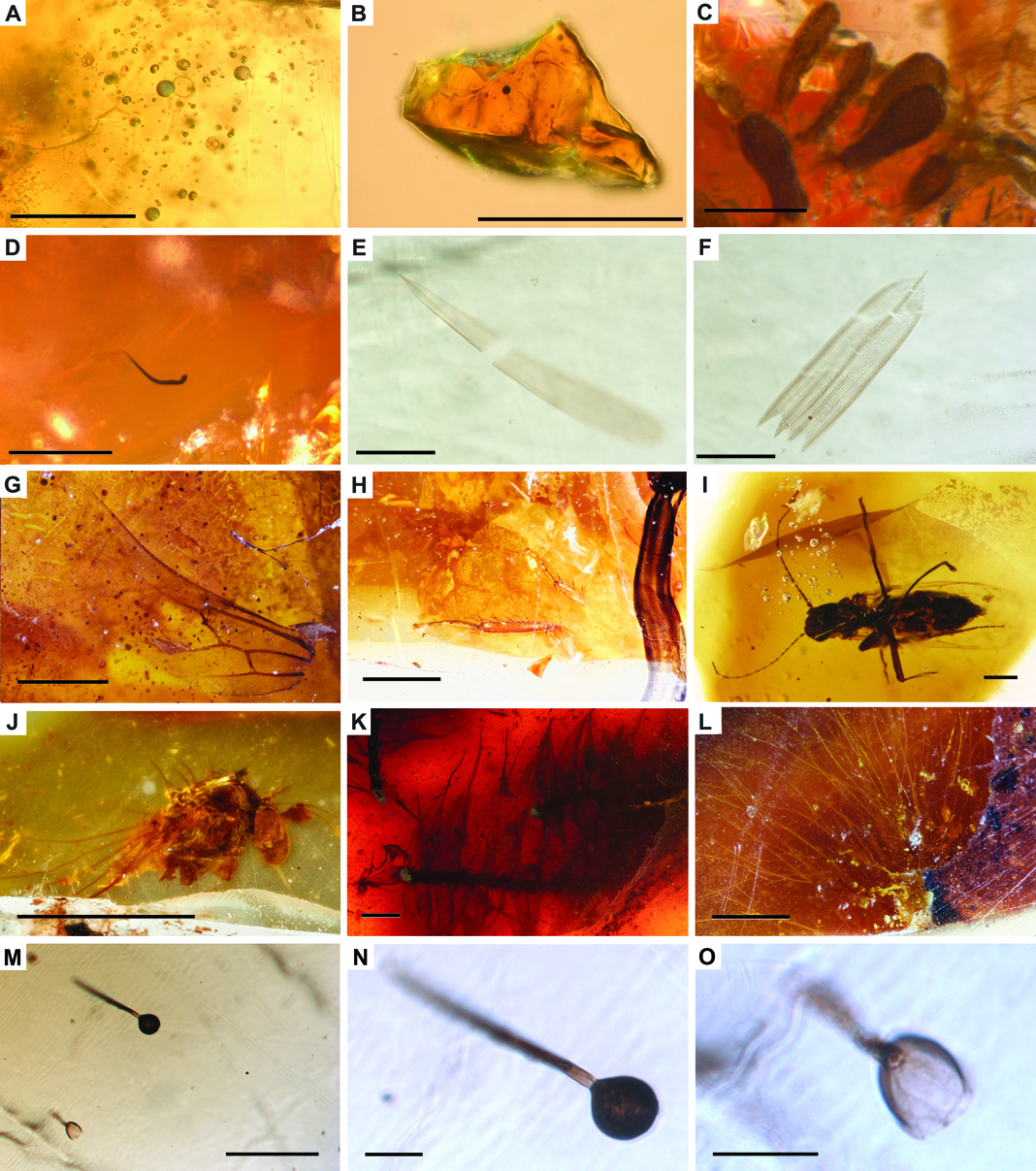


**Fig. S1. Additional assortment of amber pieces and bioinclusions from Southern Pangea and Southern Gondwana.** (**A** to **B**) Amber fragments from Fingal Coal Measures, Tasmania, Late Triassic (Carnian). (A) Inclusions, likely vacuoles/froth caused by air in exudation of resin (scale = 250 µm). (B) Amber fragment (scale = 250 µm). (**C**) Potential plant material, but rare deformed bubbles cannot be discounted, from Strahan, western Tasmania, early Eocene (scale = 200 µm). (**D**) Nematode from Strahan, western Tasmania, early Eocene (scale = 200 µm). (**E** to **O**) Bioinclusions from Anglesea amber, Victoria, late middle Eocene. (E) Elongated scale of Lepidoptera (scale = 50 µm). (F) Well preserved digitated scale of Lepidoptera (scale = 50 µm). (G) Hind wing of ant (scale = 1 mm). (H) Cockroach legs (scale = 1 mm). (I) Ceratopogonidae specimen or biting midge of the genus *Meunierohelea* (scale = 0.2 mm). (J) Incomplete Dolichopodidae specimen (scale = 1 mm). (K) Multiple stems with perfectly preserved phyllids or leaf-like structures of mosses of the genus *Racopilum* (Bryophyta: Racopilaceae) (scale = 0.5 mm). (L) Fungal mycelia (scale = 1 mm). (M to O) Hyphomycete similar to the extant anamorphic genus *Monotosporella*, which has a previous fossil record in lower Eocene Indian amber (*60*), with detail of a single conidium (O) (scales = 0.1 mm, M, 20 µm, N and O).


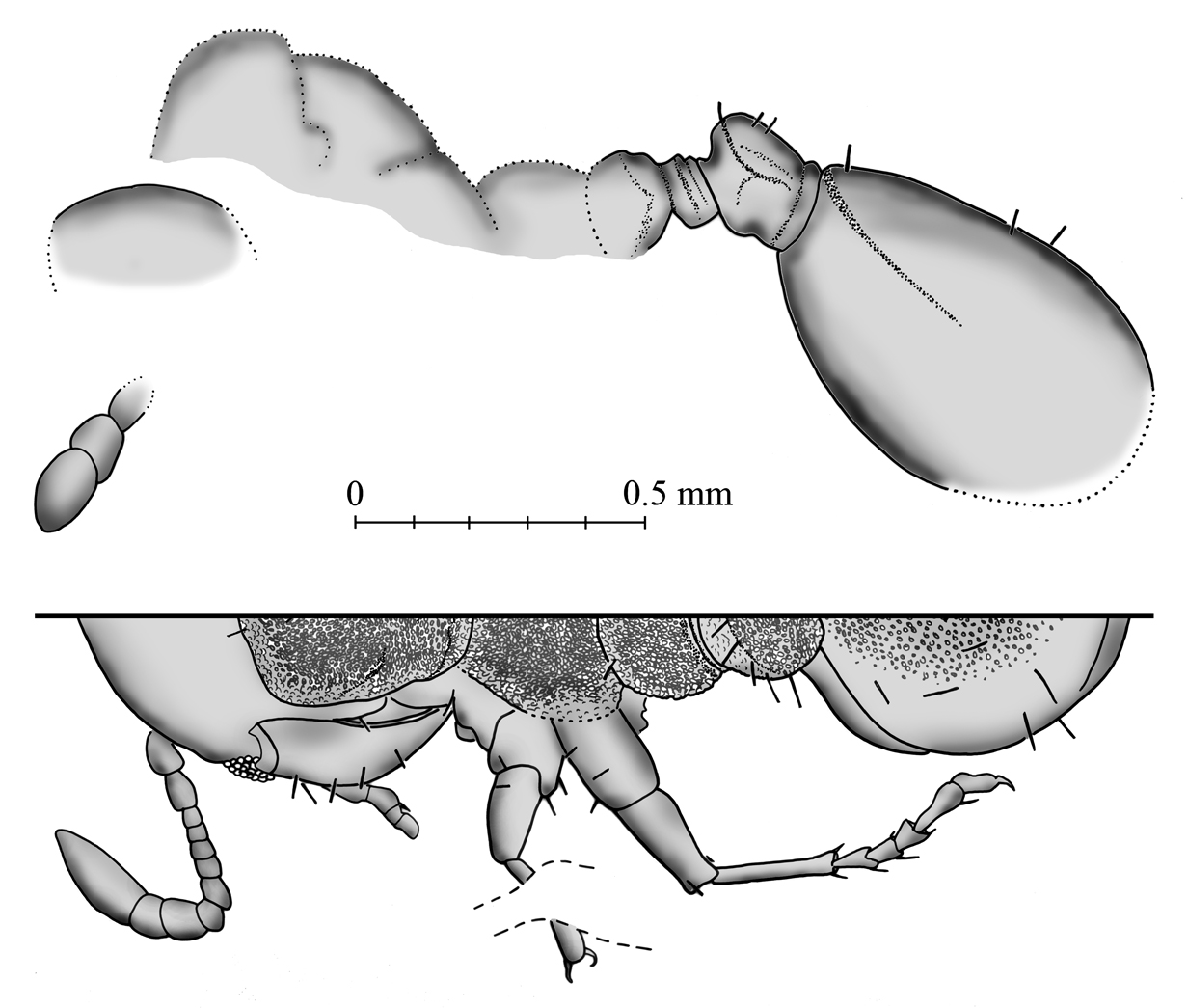


**Fig. S2. Camera lucida drawings of a complete worker ant of the living genus *Monomorium* or a "*Monomorium*-like" lineage (Hymenoptera: Formicoidea: Formicidae) from the late middle Eocene amber of Anglesea.** (**A**) Lateral view. (**B**) Left half of the dorsal view at the same scale. Figure performed using an Olympus U-DA drawing tube attached to the Olympus BX51 compound microscope and by using Adobe Photoshop software (CS2, version 9.0; www.adobe.com).
